# Imaging of Accidental Contamination by Fluorine-18 Solution: A Quick Troubleshooting Procedure

**DOI:** 10.7508/aojnmb.2016.04.008

**Published:** 2016

**Authors:** Kalevi Kairemo, Aki Kangasmäki

**Affiliations:** 1 Department of Molecular Radiotherapy & Nuclear Medicine, Docrates Cancer Center, Helsinki, Finland; 2 Department of Radiation Physics, Docrates Cancer Center, Saukonpaadenranta, Helsinki, Finland

**Keywords:** F-18, Quantitative gamma imaging, Radio fluorine uptake, Radiopharmaceutical preparation, Skin contamination

## Abstract

To the best of our knowledge, imaging of accidental exposure to radioactive fluorine-18 (F-18) due to liquid spill has not been described earlier in the scientific literature. The short half-life of F-18 (t_½_=110 min), current radiation safety requirements, and Good Manufacturing Practice (GMP) regulations on radiopharmaceuticals have restrained the occurrence of these incidents. The possibility of investigating this type of incidents by gamma and positron imaging is also quite limited. Additionally, a quick and precise analysis of radiochemical contamination is cumbersome and sometimes challenging if the spills of radioactive materials are low in activity. Herein, we report a case of accidental F-18 contamination in a service person during a routine cyclotron maintenance procedure. During target replacement, liquid F-18 was spilled on the person responsible for the maintenance. The activities of spills were immediately measured using contamination detectors, and the photon spectrum of contaminated clothes was assessed through gamma spectroscopy. Despite protective clothing, some skin areas were contaminated, which were then thoroughly washed. Later on, these areas were imaged, using positron emission tomography (PET), and a gamma camera (including spectroscopy). Two contaminated skin areas were located on the hand (9.7 and 14.7 cm^2^, respectively), which showed very low activities (19.0 and 22.8 kBq respectively at the time of incident). Based on the photon spectra, F-18 was confirmed as the main present radionuclide. PET imaging demonstrated the shape of these contaminated hot spots. However, the measured activities were very low due to the use of protective clothing. With prompt action and use of proper equipments at the time of incident, minimal radionuclide activities and their locations could be thoroughly analyzed. The cumulative skin doses of the contaminated regions were calculated at 1.52 and 2.00 mSv, respectively. In the follow-up, no skin changes were observed in the contaminated areas.

## Introduction

Accidental exposure to radioactive fluorine-18 (F-18) has not been described earlier in the scientific literature. The short half-life of F-18 (t_½_=110 min), radiation safety requirements, and Good Manufacturing Practice (GMP) regulations on radiopharmaceuticals have restrained the occurrence of these incidents. However, minor contaminations are still registered in the routine clinical use of F-18 (1).

Although the service personnel should follow radiation safety principles, they do not always act with GMP regulations in mind. On the other hand, quick and precise analysis of radiochemical contamination is cumbersome and sometimes challenging if the spills of radioactive materials are low in activity.

Surface contamination monitors are used to locate the contaminated areas, measure contamination levels, and monitor the success of decontamination procedures. While contamination monitors are excellent for radiation protection purposes, they cannot be used effectively to assess spill patterns or to gain knowledge of the precise spatial location of the most active spots. An image scanner, gamma camera, or positron emission tomography (PET) camera can be employed to observe the spatial distribution of contaminated areas. When used in combination, both contamination activity and spatial distribution can be determined.

Herein, we report a case of contamination where prompt action allowed for the careful analysis of skin contamination with low activity.

## Case report

A 34-year-old male worker responsible for cyclotron maintenance was exposed to liquid contamination, while replacing the targets of PET-trace 10 cyclotron (General Electric, Fairfield, CT, USA) during routine maintenance procedure. Although the worker wore a protective overall, use of protective gloves during the maintenance work was not possible. The liquid solution was assumed to be radioactive; therefore, the worker immediately washed his hands carefully. Afterwards, a surface contamination detector (Rados, RDS-80, Turku, Finland) was used, and the measurements were performed within 20 min following the incident.

The background activity was 15 cps on average. Radionuclide activity was observed on the worker’s hands and clothes. The detailed activities were as follows: left thumb, 400-430 cps; left index finger, 220 cps; left middle finger, 20 cps; left fingers IV-V, 10 cps; all right hand fingers, < 20 cps; chest, 2000 cps; pelvis, 1500 cps; and shoes, 500 cps (Table 1). The shoes and clothes were changed and measured separately via gamma spectroscopy in order to identify the radionuclides. No other radionuclides were detected besides F-18.

**Table 1:** Contamination measurements with a hand-held detector at 20 and 90 min following the incident

Measured organs	Activity (cps) at 20 min	Activity (cps) at 90 min
Left hand: thumb	400-430	Both hands 475
Left hand: index finger	220	
Left hand: middle finger	20	
Left hand: IV & V fingers	<10	Wrist 95-125
Left hand: volar	10	
Left hand: palmar	70	
Right hand: thumb	<20	Both hands 10
Right hand: index finger	<10	
Right hand: middle finger	<10	
Right hand: IV & V fingers	<20	
Right hand: volar	20	
Right hand: palmar	20	
Thorax	2000	
Pelvis	1500	10
Shoes	500	
Body elsewhere	<20	
Background	10-20	10

The hands were once again washed thoroughly until no change in radionuclide activity was detected in contamination measurements of the left hand. Measurements at 90 min following the incident are presented in Table 1.

The Nuclear Medicine Department was informed about the incident and the person went through special scanning procedures.

**Figure 1:**
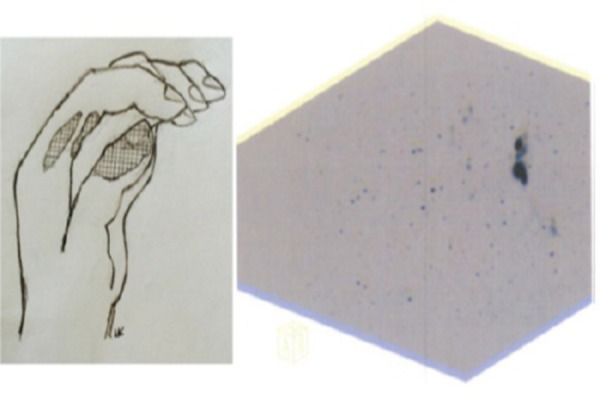
Three-dimensional PET study of the left hand (on the right) and schematic illustration of the contamination site in fingers I & II (grid pattern). There is activity in the distal part of finger I and proximal parts of finger II (palmar & radial surfaces). In the PET image, the lower hot spot corresponds to the activity in finger I, and the activity above it demonstrates the two areas of proximal finger II. The total F-18 activity was less than 50 kBq at the time of imaging, and therefore, the acquisition time was 15 min. Note that the fist is more opened in the schematic view than the actual PET image, but still the regions can be distinguished

His left hand was scanned, using Siemens Biograph PET Camera, and two separate hot spots were found in the regions of fingers I and II, as shown in Figure 1. Another active site was found in the wrist region. This contamination was only found in nuclear imaging procedures and may have originated from the hand washing process.

**Figure 2:**
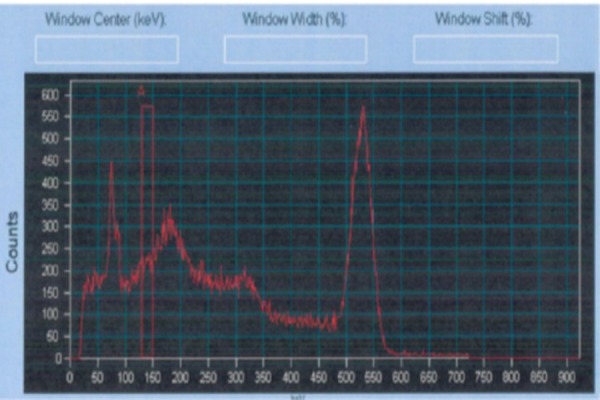
The gamma spectrum measurement of the contamination demonstrates an energy peak in 500-550 keV region (due to positron formation at 511 keV). The rectangular bar shows the energy window used for Co-57 marker in the superimposed image (Figure 3 on the right)

The regions were scanned by another surface contamination detector, based on plastic scintillation detector (CoMo 170, GRAETZ Strahlungsmeßtechnik GmbH, Altena, Germany). The activity was estimated at 3-4 Bq/cm^2^ (i.e., 600 Bq) in the wrist region, which was larger, and 15 Bq/cm^2^ in the finger region (i.e., 2250 Bq) (170 cm^2^ measurement area). Afterwards, the gamma camera (Siemens Symbia T2 camera) measurements were performed on the subject’s hands. The gamma spectrum demonstrated an energy peak within the 500-550 keV region due to positron formation at 511 keV (Figure 2); therefore, the liquid was considered to contain F-18 (t_½_ =110 min).

The worker’s both hands were then placed on a contamination towel on a high-energy collimator for scanning; the acquisition time was set at 15 min. His hands were also scanned, using a low-energy collimator, and hand contours were drawn on the image, using a Co-57 marker. The images were superimposed, based on camera coordinates and the drawn hand contours on the contamination towel.

**Figure 3:**
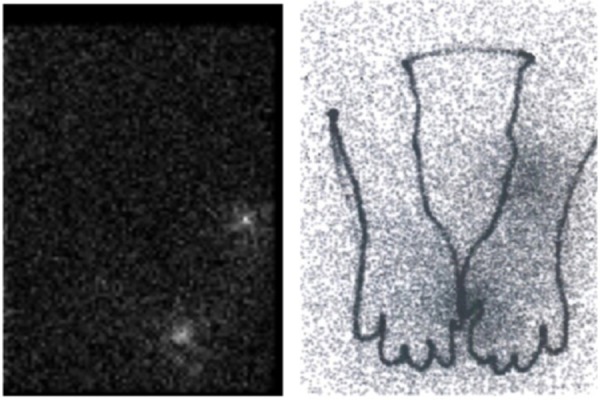
The gamma camera image using high-energy collimator revealed two hot spot regions, measured at approximately 9.7 cm^2^ (combined area of fingers I-II) and 14.7 cm^2^ (wrist), respectively, based on the number of active pixels (on the left). The superimposed image (Co-57 marker pen drawing of the upper arm contours) and the actual F-18 measurement indicate the approximate location of hot spots. The images were superimposed, based on camera coordinates and the drawn hand contours on the contamination towel

The image provided by the gamma camera revealed two hot spot regions, measured to be approximately 9.7 cm^2^ (surface area of fingers I & II) and 14.7 cm^2^ (surface area of the wrist), based on the number of active pixels, respectively (Figure 3). Since the sensitivity of high-energy collimator for F-18 was unknown prior to the incident, a separate sensitivity measurement was performed afterwards: in-contact geometry using similar imaging conditions as during contamination measurements. The sensitivity for F-18 was estimated at 2.293 cpm/kBq (84.86 cpm/µCi).

The radionuclide activities in the left hand lesions were 19.0 kBq and 22.8 kBq, respectively, and no activity was reported in the right hand, as seen in the superimposed image (Figure 3). These activities were corrected for F-18 decay during the acquisition. The imaging procedures started approximately 100 min after the incident. The total activity in the contaminated hands after the cleaning procedures was less than 100 kBq. Also, the spectroscopic measurements of clothes and shoes did not reveal any long-lived radionuclides.

## Discussion

Nuclear medicine imaging tools provide an excellent opportunity for precise quantification of PET tracer accidents. However, the short half-life of many PET radionuclides is regarded as a disadvantage. For quantification, PET requires transmission imaging correction or computed tomography (CT) acquisition. In our case, CT had to be excluded to avoid additional radiation exposure. Therefore, quantitative gamma imaging is recommended if available at the accident site.

To the best of our knowledge, this is the first report to describe the imaging of accidental F-18 contamination, which may indicate the rarity of such incidents. Active training in all parts of the world can minimize these types of incidents. In addition, multiple guidelines have been proposed in the literature for radiation safety (2-4). Considering the fact that GMP and aseptic guidelines are not followed on a daily basis, the service personnel may be more prone to contamination, compared to the personnel at nuclear medicine departments. Moreover, nuclear medicine personnel are exposed to radioactive tracers every day and only minor contaminations occur (1).

Fluorine is the most clinically used PET radionuclide, considering the various clinical applications of fluorodeoxyglucose and F-18 (5, 6). The physical half-life of F-18 is 1.83 hours and its biological half-life is approximately 6 hours, making its effective half-life approximately 1.4 hours (7). Critical organs are the lungs (inhaled F-18) and stomach (ingested F-18), and the absorbed doses for F-18 are 1.4 E-10 Sv/Bq in the lungs and 2.9 E-10 Sv/Bq in the stomach (8).

Skin dose rate conversion factors after contamination with radiopharmaceuticals, including F-18, have been reported in the literature (9). In our case, the skin doses were 1.52 and 2.00 mSv, as calculated by Monte Carlo particle transport code, i.e., approximately 1.7 mGyh^-1^kBq^-1^. The skin doses were low in our case, since F-18 contamination varies from approximately 0.02 to 20 mSv in nuclear medicine technicians (1). The contaminated worker in our case did not use a ring dosimeter during the incident; therefore, all dose estimations are crude approximations.

In the present case, the performed imaging procedures were successful, and no essential skin absorption could be detected on PET images. The three-dimensional configuration of the liquid spill was represented exquisitely on separate fingers and separate surfaces (Figure 1). The differences in activity measurements are mainly explained by geometries and time of measurements. It should be mentioned that hand-held detectors and gamma imaging procedures are both two-dimensional techniques.

Generally, severe skin contaminations can be managed, although some activity always remains; therefore, various cleaning procedures have been proposed over the decades (10, 11). Fortunately, the service worker in our case did not develop any skin changes due to the remaining radioactive spill in the follow-up.
